# Optimized and Functionalized Carvacrol-Loaded Nanostructured Lipid Carriers for Enhanced Cytotoxicity in Breast Cancer Cells

**DOI:** 10.3390/pharmaceutics17030363

**Published:** 2025-03-13

**Authors:** Ana F. C. Uchôa, Allessya L. D. Formiga, Anny L. M. R. Cardoso, Graziela M. A. Pereira, Lucas M. M. Carvalho, Pedro H. O. Souza, Anauara L. Silva, Ramon R. M. Souza, Marianna V. Sobral, Marcelo S. Silva, José M. Barbosa-Filho, Francisco H. Xavier-Júnior

**Affiliations:** 1 Laboratory of Pharmaceutical Biotechnology (BioTecFarm), Department of Pharmaceutical Sciences, Federal University of Paraiba, Campus Universitário I, Castelo Branco III-Cidade Universitária, João Pessoa 58051-900, PB, Brazil; anauchoa@ltf.ufpb.br (A.F.C.U.); allessya.formiga@ltf.ufpb.br (A.L.D.F.); annyleticiamarinho@ltf.ufpb.br (A.L.M.R.C.); graziela.maria.araujo.pereira@academico.ufpb.br (G.M.A.P.); lucas.medeiros2@academico.ufpb.br (L.M.M.C.); phos@academico.ufpb.br (P.H.O.S.); 2 Multiuser Characterization and Analysis Laboratory (LMCA), Institute of Research in Drugs and Medicines, Federal University of Paraiba, Campus Universitário I, Castelo Branco III-Cidade Universitária, João Pessoa 58051-900, PB, Brazil; anauaralima@ltf.ufpb.br (A.L.S.); marcelosobral@ltf.ufpb.br (M.S.S.); jbarbosa@ltf.ufpb.br (J.M.B.-F.); 3 Oncopharmacology Laboratory (ONCOFAR), Institute of Research in Drugs and Medicines (IPeFarM), Federal University of Paraiba, Campus Universitário I, Castelo Branco III-Cidade Universitária, João Pessoa 58051-900, PB, Brazil; ramonsouza@ltf.ufpb.br (R.R.M.S.); mariannavbs@gmail.com (M.V.S.)

**Keywords:** carvacrol, nanotechnology, drug delivery, nanostructured lipid carriers, breast cancer

## Abstract

**Background/Objectives**: Carvacrol, a monoterpenoid phenol found in essential oils, exhibits many biological activities, including anticancer properties through mechanisms such as induction of apoptosis. These properties can be enhanced if encapsulated within nanoparticles. This study focuses on producing functionalized carvacrol-loaded nanostructured lipid carriers (NLCs) applied to the treatment of breast cancer. **Methods**: NLCs were produced by hot emulsification with the sonication method and optimized by the Box–Behnken design, considering Precirol^®^ (1, 4, 7%), carvacrol (1, 5, 9%), and Tween^®^ (0.1, 0.5, 0.9%) as independent variables. **Results**: The optimized NLC containing 2% carvacrol had a particle size of 111 ± 2 nm, PdI of 0.26 ± 0.01, and zeta potential of −24 ± 0.8 mV. The solid lipid (Precirol^®^) was the variable that most influenced particle size. NLCs were functionalized with Pluronic^®^ F68, cholesterol, chitosan, and polyethylene glycol (0.05–0.2%), with oNLC-Chol presenting the most promising results, with no significant increase in particle size (±12 nm) and high encapsulation efficiency (98%). Infrared spectra confirm effective carvacrol encapsulation, and stability tests showed no significant physicochemical changes for 120 days of storage at 4 °C. When incubated with albumin (5 mg/mL), NLCs showed overall good stability over 24 h, except for oNLC-Chol, which increased slightly in size after 24 h. In addition, oNLC increased the cytotoxic effect of carvacrol by 12-fold, resulting in an IC_50_ of 7 ± 1 μg/mL. **Conclusions**: Therefore, it was possible to produce stable, homogeneous NLCs with nanometric sizes containing 2% carvacrol that displayed improved anticancer efficacy, indicating their potential as a delivery system.

## 1. Introduction

Cancer is a major global public health concern, consistently ranking among the top four causes of mortality for individuals under the age of 70 [1]. It is characterized by uncontrolled cellular proliferation, invasion of adjacent tissues, and the potential for metastasis to distant sites [2]. As a heterogeneous disease, its prevalence and outcomes are influenced by individual genetics, with tumor development requiring the accumulation of cellular mutations [3,4]. Among the types of cancer, breast cancer is the second most frequently diagnosed type (12% of cases), standing out as one of the leading causes of cancer-related mortality worldwide, being more prevalent among females and in developing countries [5].

Currently, cancer treatment varies according to the type and severity of the disease. Antineoplastic agents are the primary treatment for preventing recurrence and metastasis. However, they tend to exhibit high toxicity due to low specificity for tumor cells [6], leading to severe side effects in secondary organs, such as nausea, vomiting, anorexia, weakness, and alopecia [7]. Thus, natural products from medicinal plants can represent a promising therapeutic alternative, given their varied composition of secondary metabolites [8]. For instance, phenolic monoterpene volatile compounds, such as carvacrol, found in oregano (*Origanum vulgare* L.), confer various well-documented biological activities, including antioxidant [9], antineoplastic [10], antinociceptive [11], and anti-inflammatory properties [12].

Nevertheless, phytochemicals still face physicochemical challenges concerning solubility and distribution in the bloodstream, with the added risk of inducing toxicity in peripheral tissues. Consequently, there is growing interest in studying mechanisms that can effectively target these molecules to their specific neoplastic tissues [13,14]. Nanotechnology, for example, presents many advantages, such as the development of particles with various sizes and compositions that are capable of encapsulating bioactive molecules, thereby protecting them and targeting desired tissues [15].

Within the scope of nanotechnology, nanostructured lipid carriers (NLCs) stand out for their advantages over other types of nanoparticles, such as improved permeability and bioavailability, increased solubility, better storage stability, and reduced adverse reactions [16]. Their effectiveness is also attributed to their reduced diameter, which facilitates adsorption on the cell surface, leading to an increased concentration gradient and, consequently, improved transport of biologically active substances to inflamed tissues, enhancing pharmacokinetics and pharmacodynamics, and reducing toxicity [17].

Makeen and collaborators (2021) [18], for example, developed a study using NLCs for breast cancer treatment in an MCF-7 cell line, utilizing high-pressure homogenization to prepare particles containing the drug Imatinib. The results indicated an 8.75-fold increase in the efficacy when compared to the isolated molecule. However, there are no studies in the literature specifically addressing the encapsulation of carvacrol for this purpose. Therefore, NLCs containing this component present a promising and innovative approach to the treatment of various diseases.

Taken together, the current article is based on the characterization, functionalization, and evaluation of the cytotoxicity of a stable and biodegradable nanotechnological system capable of delivering biologically active natural molecules for potential application against breast cancer cells. Thus, carvacrol-loaded NLCs produced by ultrasonication were optimized by the Box–Behnken experimental design. They were also functionalized with Pluronic^®^ (0.5 to 2%), cholesterol (0.05 to 0.2%), chitosan (0.05 to 0.2%), and polyethylene glycol (PEG) (0.5 to 2%), and the stability was assessed at 4, 25, and 37 °C for 120 days. Then, the interaction of NLCs with albumin was determined, and their cytotoxicity was determined against breast cancer cells (MCF-7).

## 2. Materials and Methods

### 2.1. Materials

Kolliphor^®^ ELP and Medium-chain triglycerides (MCTs) were obtained from Basf Pharma (Fremont, CA, USA). Precirol^®^ ATO 5 was obtained from Gattefossé (Paramus, NJ, USA). Lipoid^®^ S-100 was purchased from Lipoid (Ludwigshafen am Rhein, Germany). Carvacrol (75%) extracted from oregano oil was furnished by Ferquima Indústria e Comércio (São Paulo, Brazil). Cholesterol was acquired from Dishman (Veenendaal, The Netherlands). Chitosan 15 kDa was purchased from Polysciences, Inc. (Warrington, PA, USA). Polyethylene Glycol (PEG) 6000 P.A. was obtained from Dinâmica Química Contemporânea^®^ (São Paulo, Brazil). Carvacrol standard, Pluronic^®^ F-68, Tween^®^ 80, Span^®^ 60, phosphate-buffered saline (PBS), bovine serum albumin (BSA), (3-(4,5-dimethylthiazol-2-yl)-2,5-diphenyltetrazolium bromide (MTT), Dulbecco’s Modified Eagle Medium (DMEM), penicillin, streptomycin, and dimethyl sulfoxide (DMSO) were acquired from Sigma-Aldrich^®^ (St. Louis, MO, USA). All materials were used as received.

### 2.2. Production of NLCs

NLCs were produced with Precirol^®^ ATO 5 (1, 4, 7%) as the solid lipid, carvacrol (1, 5, 9%) as the liquid lipid, Tween^®^ 80 (1, 3, 5%) as the hydrophilic surfactant, and Span^®^ 60 (1%) as the lipophilic surfactant. All materials were accurately weighed and added to the same bottle. The mixture was heated in a 65 °C water bath and stirred magnetically at 1000 rpm for 5 min until fully dispersed. The selected production technique was hot emulsification followed by homogenization using an ultrasonic probe sonicator (HES) [19]. Thus, the pre-homogenized mixture was subjected to ultrasonic disruption at 65 W for 120 s using a probe sonicator (Disruptor, Ultronique^®^, Indaiatuba, São Paulo, Brazil). All produced nanosystems were cooled to room temperature and stored at 4 °C until further characterization analyses.

#### Pre-Formulation Studies

Pre-formulation tests were initially conducted using Lipoid^®^ S-100, Kolliphor^®^ ELP, and Span^®^ 60 at 1% to identify the surfactant that best interacted with the formulation components, ensuring optimal oil core compaction and nanosystem stabilization. Therefore, NLCs were produced as previously described, forming three formulation patterns with fixed concentrations of Tween^®^ 80 (5%). The impact of the lipid components, including carvacrol (2–9%) and Precirol^®^ ATO 5 (1–7%), on the final formulation was evaluated.

### 2.3. Optimization of NLCs

The Box–Behnken design was utilized to optimize the formulation of NLCs. A total of 15 formulations were prepared, varying in concentration of solid lipid (Precirol^®^ ATO 5, 1–7%), liquid lipid (carvacrol, 1–9%), and hydrophilic surfactant (Tween^®^ 80, 1–5%), which served as independent variables. The average hydrodynamic particle diameter was set as the dependent variable. The selected lipophilic surfactant concentration remained fixed at 1% across all formulations, and NLCs were synthesized following the previously described method. Finally, all data analyses were performed using Statistica^®^ software.

### 2.4. Functionalization of NLCs

The optimized NLC was surface-modified with biocompatible components to control its interaction with albumin and target cells. Functionalization was performed with Pluronic^®^ (0.5, 0.1, 0.15, and 2%), cholesterol (0.05, 0.1, 0.15, and 0.2%), chitosan (0.05, 0.1, 0.15, and 0.2%), and PEG (0.5, 0.1, 0.15, and 2%). For cholesterol (oNLC-Chol) and chitosan (oNLC-Chi), these components were added directly to the pre-emulsified lipid phase before the sonication step to ensure proper incorporation. Conversely, Pluronic^®^ (oNLC-Plu) and PEG (oNLC-PEG) were replaced by Tween^®^ 80, with their concentrations appropriately adjusted. After sonication, the formulations were cooled to room temperature and stored in sealed vials.

### 2.5. Characterization of NLCs

#### 2.5.1. Particle Size Analysis

The nanosystems’ hydrodynamic diameter and size distribution were assessed through dynamic light scattering (DLS) using a Zetasizer Lab (Malvern Instruments Ltd., Malvern, UK) at 25 °C. Measurements were conducted at a fixed scattering angle of 90 degrees, with samples diluted in Milli-Q^®^ water at a 1:100 ratio before analysis. All analyses were performed in triplicate, and the results were reported as the mean hydrodynamic diameter (size distribution) and polydispersity index (PdI).

#### 2.5.2. Zeta Potential Analysis

The zeta potential (ζ) was measured using the Zetasizer Lab (Malvern Instruments Ltd., Malvern, UK) at 25 °C. To ensure a consistent ionic strength, samples were diluted at a 1:100 ratio in a saline solution (NaCl, 1 mM) before analysis. The final results represent the average of three independent measurements.

#### 2.5.3. Fourier-Transform Infrared Spectroscopy (FTIR)

Infrared analyses were conducted on carvacrol, each component, and the final formulations using a Fourier-transform infrared spectrophotometer equipped with attenuated total reflection (FTIR-ATR, Shimadzu Cary 630, Kyoto, Japan). Data acquisition was performed within the spectral range of 4000 to 400 cm^−1^, with a resolution of 4 cm^−1^, and 64 scans recorded at 25 °C.

#### 2.5.4. Morphological Analysis of NLCs

The morphology of the nanoparticles was examined using Scanning Electron Microscopy (SEM) with a TESCAN MIRA3 system (Brno, Czech Republic). The formulations were diluted at a 1:10 ratio, and a 5 μL aliquot was placed onto a fixed slide mounted on a stub with carbon tape. Following air drying in a desiccator, the samples were coated with a thin layer of gold using a DESK II metallizer (Denton Vacuum, Moorestown, NJ, USA). Imaging was performed using an SEM at an acceleration voltage of 20 kV and a working distance of 15 mm.

### 2.6. Quantification and Encapsulation Efficiency (EE%)

To accurately quantify the carvacrol encapsulated within the carriers, the oil content and encapsulation efficiency (EE%) were assessed using calibration curves established via High-Performance Liquid Chromatography (HPLC). Carvacrol standards were prepared at concentrations of 1, 5, 10, 15, and 20 μg/mL and injected into the system (Supplementary Materials Figure S1). For the analysis, a mobile phase consisting of methanol and water in an 80:20 *v*/*v* ratio was used, which was filtered and degassed before use to ensure purity and prevent bubble formation in the system. A C18 column (250 mm × 4.6 mm, 5 μm) was utilized, with the UV detector set to a wavelength of 276 nm. The flow rate was maintained at 1.4 mL/min, and a 20 μL sample was injected for analysis. Chromatograms were recorded in triplicate, and the corresponding peak areas for each concentration were analyzed to determine the results.

Carvacrol was quantified using methanol to dilute the samples, following an ultrasonic bath at 45 °C during 20 min and centrifugation afterwards (14,000 rpm, 5 min) (Centrifuge 5430, Eppendorf^®^, Hamburg, Germany). The supernatant was collected, filtered through 0.22 μm, and analyzed as previously described. Each measurement was performed in triplicate to ensure accuracy. The EE% was assessed through ultrafiltration using Corning^®^ Costar^®^ Spin-X^®^ centrifugal filter tubes (cutoff: 14 kDa) with a 500 μL capacity. The samples were subjected to centrifugation at 5000 rpm for 15 min, after which the filtrate was collected for further analysis. The result was calculated through the equation EE% = (Total compound added − filtrate compound/Total compound added) × 100.

### 2.7. Stability Analysis

The formulations (oNLC, oNLC-Plu0.5%, oNLC-Chol0.1%, and oNLC-PEG0.5%) underwent long-term stability assessments under controlled temperature conditions. NLCs were stored in hermetically sealed vials and monitored for 120 days at 4, 25, and 37 °C. Evaluations included measurements of particle size, polydispersity index, and zeta potential, following the previously described methods.

### 2.8. Protein Corona Evaluation

The formation of the protein corona around NLCs upon binding to serum albumin was also investigated. Bovine serum albumin (BSA) was prepared in a 1 mM phosphate-buffered saline (PBS) solution at four different concentrations (1, 5, 10, and 20 mg/mL). Each final NLC sample was incubated at a 1:10 ratio and immediately analyzed for particle size and zeta potential, following previously described procedures. Additionally, to assess the temporal pattern of hard or soft corona formation, the same analyses were conducted at a fixed albumin concentration (5 mg/mL) over different time intervals (0.25, 0.5, 1, 2, 4, and 24 h).

### 2.9. Cytotoxicity Assay

The MCF-7 breast adenocarcinoma cell line and the MCF-10 non-tumoral cell line derived from normal human breast epithelial tissue were used to evaluate the cytotoxicity of the NLCs. Cells were cultured in Dulbecco’s Modified Eagle Medium (DMEM) supplemented with 10% bovine serum albumin (BSA) and 1% antibiotic solution (containing 100 U/mL of penicillin and 100 μg/mL of streptomycin). Cultures were maintained at 37 °C in a humidified incubator (CytoGROW GLP Panasonic^®^, Osaka, Japan) with 5% CO_2_. The MTT (3-(4,5-dimethylthiazol-2-yl)-2,5-diphenyltetrazolium bromide) assay was used to quantify cell viability and proliferation. For the experiment, 100 μL of cells were seeded into 96-well plates (1 × 10⁵ cells/mL). After 24 h, oNLC, oNLC-Plu0.05%, oNLC-Chol0.1%, and oNLC-PEG0.5% samples were diluted in a culture medium at different concentrations (2–25 μg/mL). Additionally, oNLC encapsulating MCT, without carvacrol (oNLC MCT), was tested. Carvacrol was solubilized in DMSO and diluted in culture medium at various concentrations (19–300 μg/mL). Cells were incubated for 24 h at 37 °C in 5% CO_2_.

Posteriorly, 110 μL of the supernatant was removed, and 10 μL of the MTT solution (5 mg/mL in PBS) was added. The plates were incubated for 4 h, and 100 μL of 10% sodium dodecyl sulfate (SDS) was utilized to solubilize the formazan crystals overnight. Absorbance was measured at 570 nm using a microplate reader (BioTek Instruments, Sinergy HT, Winooski, VT, USA). Three independent experiments were performed, each in triplicate. Data is expressed as the mean ± standard error of the mean. Statistical analysis was conducted using one-way analysis of variance (ANOVA), followed by Tukey’s test. Results were considered statistically significant at *p* < 0.001. The concentration inhibiting 50% of cell viability (IC_50_) was estimated through nonlinear regression.

### 2.10. Statistical Analysis

All experiments were conducted in triplicate, and the results are presented as mean ± standard deviation. Data from the experimental design were analyzed using analysis of variance (ANOVA) to assess the significance of variables and their interactions. Additionally, regression models, *t*-tests, and F-tests were applied, with statistical significance set at *p* < 0.05. Data analysis was performed using GraphPad Prism 8.0.2 (La Jolla, CA, USA) and Statistica 10 software (StatSoft, Inc., Tulsa, OK, USA).

## 3. Results and Discussion

### 3.1. Pre-Formulation Studies

Initially, three different NLC formulations were produced to determine the most suitable surfactant, as the type and concentration of the surfactant used significantly impacted the formation of the initial emulsion and the consequent reduction in particle size [20]. Table 1 shows the physicochemical parameters of the NLCs produced with different surfactants and lipid concentrations. NLCs stabilized by Lipoid^®^ S-100 displayed a white macroscopic appearance, though slightly flocculated with minimal bluish reflection. In contrast, formulations produced with Kolliphor^®^ ELP or Span^®^ 60 were homogeneous, exhibiting a white, milky appearance and a strong bluish reflection, suggesting smaller particle sizes.

All NLCs produced exhibited particle sizes smaller than 310 nm, with an increase in lipid components tending to enlarge particle size. According to Danaei et al. (2018) [21], particle size is the main physicochemical attribute influencing cell uptake via endocytosis. Given that tumor vasculature differs significantly from that of normal tissues, carriers of approximately 200 nm are ideal for the passive delivery of active compounds and their accumulation and retention in tumor tissue.

Regarding the polydispersity index (PdI), only nanosystems stabilized by Span^®^ 60 were suitable for the delivery of bioactive molecules via lipid systems, with PdI values up to 0.25. PdI values below 0.3 indicate homogeneous monodisperse systems [21,22]. Finally, the zeta potential, indicating the degree of repulsion between charged particles in dispersion, was generally acceptable across all formulations. Higher particle charges (>|20| mV) reduce the likelihood of aggregation and particle size increase due to electrical repulsion [21].

Therefore, Span^®^ 60 was chosen as the lipophilic surfactant for optimizing NLC production, as it produced smaller particles and systems with better polydispersity and zeta potential compared to the other surfactants. This is possibly due to better chemical interaction between Span^®^ 60 and the lipid components, leading to improved carvacrol encapsulation and solid lipid compaction.

### 3.2. Optimization of NLCs

After selecting the most suitable surfactant for the NLCs, a Box–Behnken experimental design was developed, involving 15 randomized formulations, to achieve an optimized formulation with reduced particle size and good stability, homogeneity, and oil encapsulation capacity. All 15 nanocarriers presented a white, milky macroscopic appearance and a bluish reflection, characteristic of nanometric-scale colloids. The hydrodynamic diameter range of the particles varied from 88.1 to 323 nm, with an average PdI of 0.26 and zeta potentials > |20| mV (Table 2). Thus, the particle sizes, PdI, and zeta potential were suitable for the targeted delivery of actives to tumors, as reported by [21,23].

**Figure 1:**
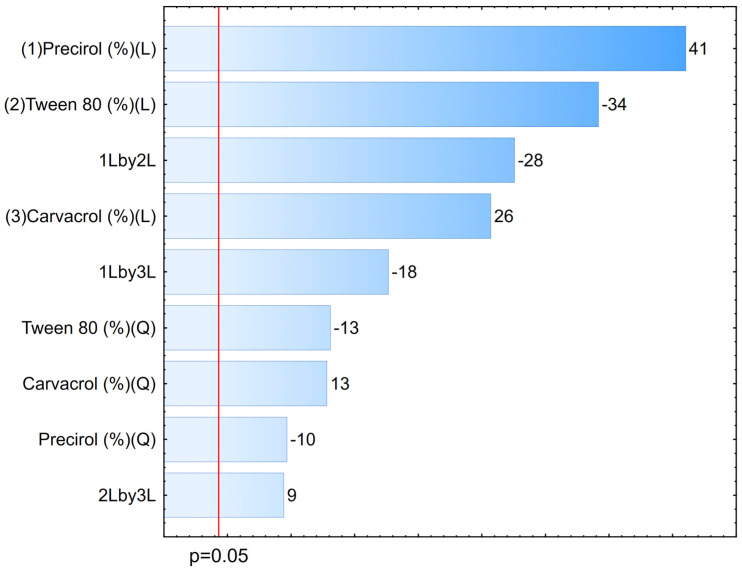
Pareto diagram obtained from the optimization of NLCs production.

**Figure 2:**
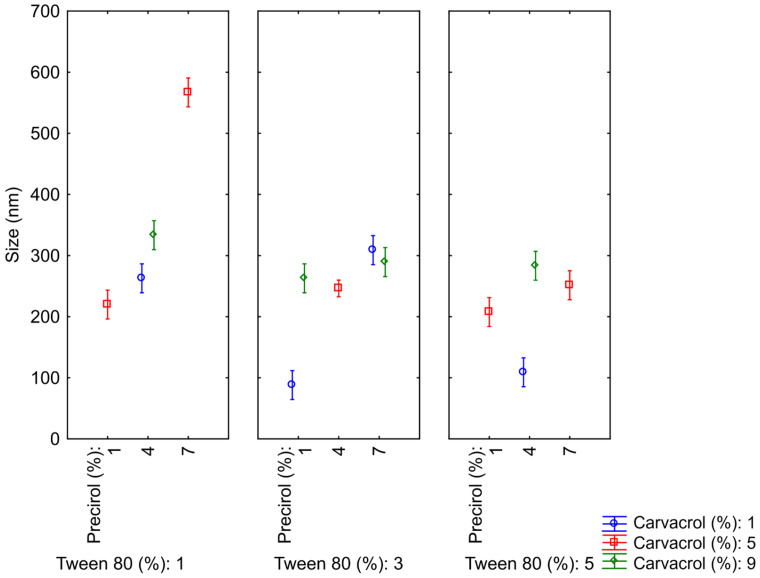
Marginal means and confidence limits of the independent variables used in the experimental design.

**Figure 3:**
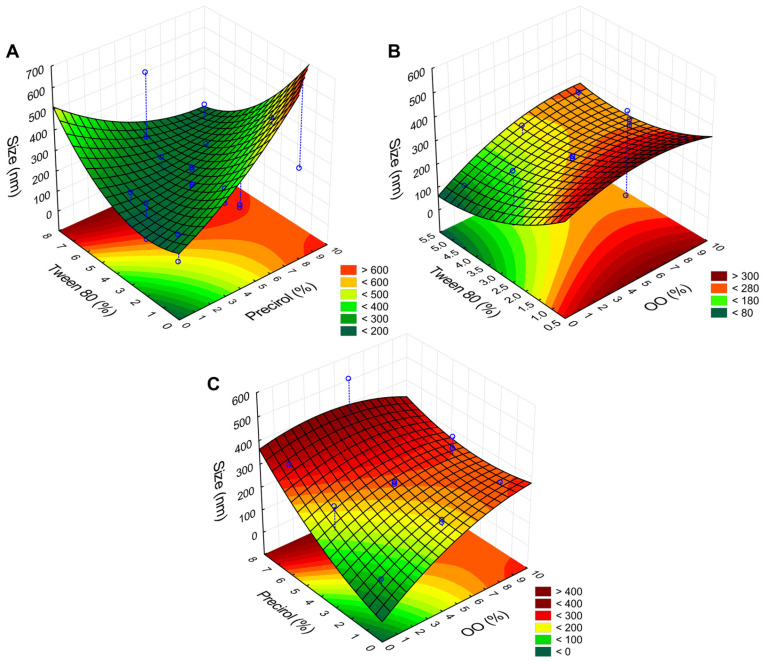
Response surface of independent variables concerning particle size. (**A**) Interaction between Tween and Precirol; (**B**) Interaction between Tween and carvacrol; (**C**) Interaction between Precirol and carvacrol.

To assess the significance of the variables and their interactions in the design, an analysis of variance (ANOVA) was conducted. The coefficient of determination (R^2^) for the size analysis was 0.97, which is considered statistically significant, as it is close to 1, indicating a good correlation between the linear and quadratic interaction models and the produced NLCs. Furthermore, to estimate how and to what extent each variable affects particle size, Statistica^®^ software was used to generate Pareto diagrams (Figure 1), confidence limit graphs (Figure 2), and response surface plots (Figure 3), analyzing both the individual effects of the variables and their interactions.

In the Pareto diagram, the length of each bar represents the standardized effect of the variable—or the interaction between two variables—and its correspondence to the response, while the signs of each variable, to the right of the bars, indicate whether the effect is favorable or unfavorable to the expected response. Positive values indicate that the variable contributes to an increase in particle size, while negative values indicate a reduction. The letters “Q” and “L” represent the quadratic and linear behaviors of the variables, respectively. All three variables—carvacrol, Tween^®^ 80, and Precirol^®^ ATO 5—are significant in both the quadratic and linear models, meaning they directly affect the average particle size obtained. On one hand, the variables or interactions that most influenced the reduction in the analyzed parameter were Tween^®^ 80, the Precirol^®^–Tween^®^ 80 interaction, and the carvacrol–Precirol^®^ interaction, all linearly. On the other hand, Precirol^®^ and carvacrol were the variables that most influenced the increase in particle size.

The variable with the most significant effect on the size parameter, as shown in the diagram, was the solid lipid Precirol^®^ ATO 5. In this study, increasing its concentration in the systems also increased the particle diameter, leading to the formation of less homogeneous nanocarriers, possibly due to the incomplete compaction of the solid by the liquid and oily components [24,25]. Higher concentrations of carvacrol also seemed to contribute negatively, possibly by saturating the system and preventing its complete encapsulation. Conversely, the reduction in particle size was primarily induced by increasing the concentration of the hydrophilic surfactant Tween^®^ 80, which supports findings by Silva et al. (2021) [26], where the presence of the surfactant in any colloid is not only essential but also contributes to the formation of a stable dispersed system, significantly reducing particle size.

The confidence limit graphs illustrate the interaction between the three variables and their consequent influence on particle size (Figure 2). It is observed that as the concentration of Tween^®^ 80 increases, it becomes possible to vary the concentration of solid lipid over a wider range. At the lowest concentration (1%), there is a significant disparity in diameters when altering the proportion of Precirol^®^, a fact that is not observed when Tween^®^ 80 is at 5%, resulting in nanocarriers of similar sizes. This once again emphasizes the importance of the hydrophilic surfactant in maintaining the size and homogeneity of the system, as previously described.

Additionally, the response surface plots below (Figure 3) were produced to confirm the proposed theories regarding the interactions between components and their influences on size. Figure 3A,B, representing the interaction of Precirol^®^ and carvacrol with Tween^®^ 80, shows smaller particle sizes when the surfactant is present in higher quantities, as expected. However, the Tween^®^–carvacrol interaction appears to be more sensitive to variations, as even minimal carvacrol concentrations require the surfactant to exceed 2% to stabilize the system. Figure 3C shows the interaction between the lipids, which seems favorable up to a concentration of about 4% of each.

As discussed, the formulation with the smallest particle size was composed of 1% carvacrol, 1% Precirol^®^, 3% Tween^®^ 80, and 1% Span^®^ 60. However, alongside the small hydrodynamic diameter of its particles (88 nm), there was also a lower amount of encapsulated oil, leading to optimization for the production of four additional similar systems (Supplementary Materials Figure S2), with gradual increases in carvacrol concentration (2, 3, 4, and 5%, respectively), since this would be the most important and bioactive component of the NLCs. As a result, homogeneous systems with particle sizes ranging from 111 to 244 nm, PdI 0.24 to 0.28, and zeta potentials of −24 to −33 mV were produced. As expected, increasing the concentration of carvacrol without altering the surfactant concentration led to an increase in particle size, as discussed through the response surface plots.

Therefore, since the final goal of the tests was to select formulations with minimal sizes, the study was continued with the optimized formulation 8A, onwards referred to as oNLC, which contained 2% carvacrol, with an average size of 111 ± 2 nm, PdI of 0.26 ± 0.01, and ζ of −24 ± 1 mV, meeting the expected quality standards (small particle size, PdI around 0.25, and ζ > |20| mV) and falling within the ideal range for efficient cellular uptake.

### 3.3. Functionalization of NLCs

**Figure 4:**
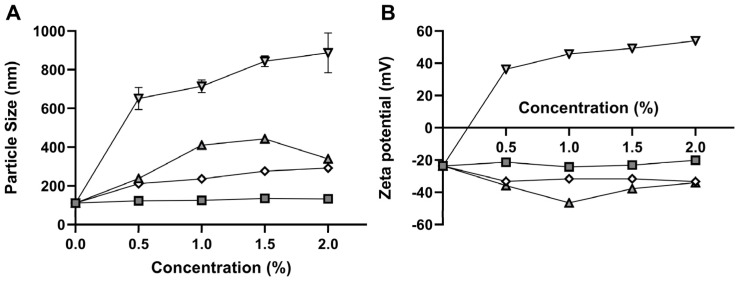
Effect of the component concentrations on (**A**) particle size and (**B**) zeta potential during NLC production. oNLC-Plu (**−Δ−**), oNLC-Chol/10 (**−☐−**), oNLC-Chi/10 (**−∇−**), and oNLC-PEG (**−◊−**).

To improve the optimized system (oNLC), functionalization was performed. This process involved replacing or adding biocompatible materials to the surface of the particles. Figure 4 illustrates the variation in size, PdI, and zeta potential with the concentration increase of each functionalizing component.

The oNLC-Chi functionalization induced the most significant size increase, with a proportional rise in particle diameter (from 651 to 887 nm) as concentration increased. This effect may result from poor interaction between chitosan and carvacrol, leading to chitosan accumulation outside the nanoparticles and consequent aggregation. This behavior is attributed to the formation of hydrogen bonds between the amine groups in the chitosan structure, which makes the system less homogeneous and more unstable, as previously described by Vinsova and Vavrikova (2008) [27]. Chitosan, a biocompatible, biodegradable, mucoadhesive, and non-toxic cationic polysaccharide, is frequently employed in nanocarrier systems for drug delivery [28]. In this work, it was utilized to enhance the mucoadhesive properties of NLCs for potential biological applications, as polymeric coatings can improve the oral bioavailability of bioactive molecules while also stabilizing the system by functioning as a surfactant [29]. Additionally, chitosan improves NLC absorption and stability in the gastrointestinal tract and prevents drug leakage, thereby increasing encapsulation efficiency [30].

The addition of Pluronic^®^, although also causing a size increase, had a smaller effect—just over 100 nm—indicating less system compaction compared to the optimized NLC containing Tween^®^ 80. Pluronic^®^ induced size variations from 238 to 340 nm and significant changes in PdI values, which reached 0.55, indicating a more polydisperse system. This may be attributed to lower compatibility with the lipophilic surfactant used in the formulation [31] and a reduced capacity to disperse the encapsulated oil [32]. Pluronic^®^ F-68 is an anionic, biocompatible polymer used as an alternative surfactant to compact systems, aiming for smaller, more stable particles with higher zeta potential and reduced particle aggregation [33]. The PEG chains in Pluronic^®^ also provide stealth properties to the nanocarriers, potentially delaying their clearance from the bloodstream [34].

Cholesterol functionalization resulted in a more homogeneous system than the original, with PdI values ranging from 0.21 to 0.23, and no significant increase in particle size (123 to 133 nm). Cholesterol was incorporated to promote interactions with the phospholipid bilayers of target cells, thereby improving the internalization of the encapsulated contents and increasing the compactness of the oily core within the NLC, filling gaps in its matrix to create smaller homogeneous particles [35,36]. Additionally, cholesterol could improve oil encapsulation efficiency by reducing lipid fluidity, providing enough rigidity to prevent leakage [37]. This coating may also increase cellular uptake and inhibit cancer cell proliferation [38]. As discussed by Karn-Orachai (2014) [39], cholesterol-coated carriers tend to reduce particle size by enhancing solid lipid–oil mixing and maintaining colloidal repulsion. Gardouh et al. (2018) [40] also demonstrated increased colloidal stability and reduced particle aggregation.

Finally, PEG, a widely used polymer in nanomedicine, can prolong circulation time and improve drug efficacy [41]. PEGylation prevents nanoparticle recognition and elimination by the innate immune system, protecting the surface from aggregation, opsonization, and phagocytosis, thus extending systemic circulation and improving drug delivery efficiency [42,43]. PEG-coated nanoparticles also showed a size increase proportional to concentration (211 to 292 nm), while PdI significantly increased (0.22 to 0.43), indicating reduced system homogeneity.

**Figure 5:**
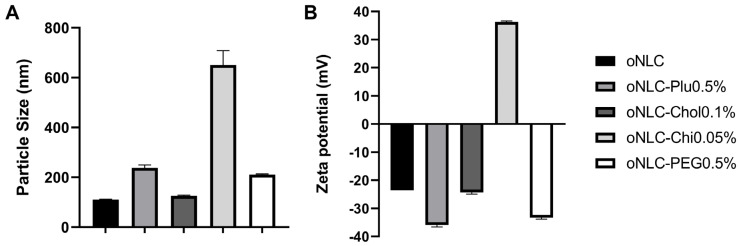
Variation of particle size (**A**) and zeta potential (**B**) induced by each final functionalized NLC.

Regarding zeta potential, all nanocarrier systems demonstrate satisfactory results, with values above |20| mV (Figure 5B). oNLC-Chi, coated with amine-rich material [44], exhibited a positive charge, ranging from +36 to +54 mV. The other functionalization showed negative results due to the hydroxyl (OH–) groups in their chemical structures, with values ranging from −36 to −47 mV for Pluronic^®^, −20 to −24 mV for cholesterol, and −32 to −33 mV for PEG. Figure 5 presents the physicochemical parameters of functionalized formulations at the lowest concentrations of each functionalizing component (oNLC-Chol 0.05%, oNLC-Plu 0.5%, oNLC-Chi 0.05%, and oNLC-PEG 0.5%).

### 3.4. Fourier-Transform Infrared Spectroscopy (FTIR)

**Figure 6:**
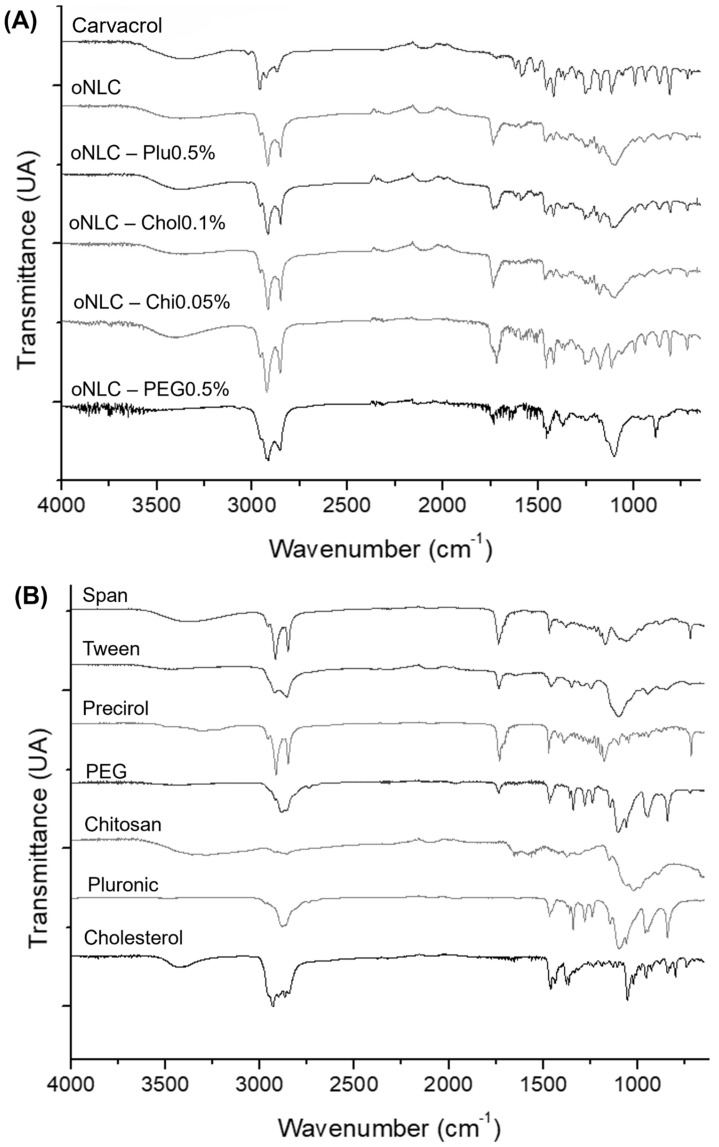
Infrared spectra of formulations (**A**) and each individual component (**B**).

Infrared spectra were conducted to chemically confirm the components used and the formulations produced. Carvacrol (Figure 6A) showed a broad band around ~3400 cm^−1^, corresponding to O-H stretching vibrations. The three bands present between 2800 and 2980 cm^−1^ were attributed to C–H stretching vibrations, arising from aliphatic CH_2_– groups, due to angular deformations around ~1450 cm^−1^. Furthermore, near 1500 cm^−1^, C=C double bond stretching from the aromatic rings of carvacrol and thymol could be detected, and between 1200 cm^−1^ and 1000 cm^−1^, several bands were attributed to C–O–H stretching vibrations. These are characteristic of carvacrol and thymol, as they possess a hydroxyl group attached to a carbon on the aromatic ring, generating C–O bonds [45,46].

The encapsulation of carvacrol in the NLCs can be confirmed by the suppression of some of its main bands, including ~2800, 1500–1600, 1250, 1000, and 600 cm^−1^. This suggests that carvacrol is encapsulated within the NLC matrix, as it cannot be detected with the same intensity on the surface. oNLC-Plu1% displayed CH_2_ stretching in the 1250 cm^−1^ region, a distinctive feature of Pluronic^®^, as well as a peak around 1100 cm^−1^ indicative of ether groups in its chemical structure [47]. oNLC-Chol0.05% showed intensified aliphatic CH stretching in the 2850 cm^−1^ region, along with the appearance of CH_2_ bending around 1450 cm^−1^, indicating cholesterol binding to the particle surface [48].

A broad band was observed at 3300 and 3500 cm^−1^, indicating the presence of secondary amides in oNLC-Chi0.1%, confirming the presence of chitosan on its surface [49]. The appearance of signals at 1653 cm^−1^ and 1620 cm^−1^, corresponding to the stretching of these groups, further confirms this presence. Meanwhile, oNLC-PEG1% showed enhanced intensity bands in 2910 cm^−1^ and strong vibrations around 1100 cm^−1^, corresponding to C–H and C–O bonds of the ether groups in the PEG chemical structure [50]. This analysis not only confirmed the efficient encapsulation of carvacrol within the NLCs but also validated the successful functionalization with the selected materials, highlighting the structural stability and compatibility of the formulations and underscoring their potential as delivery systems.

### 3.5. Scanning Electron Microscopy (SEM)

**Figure 7:**
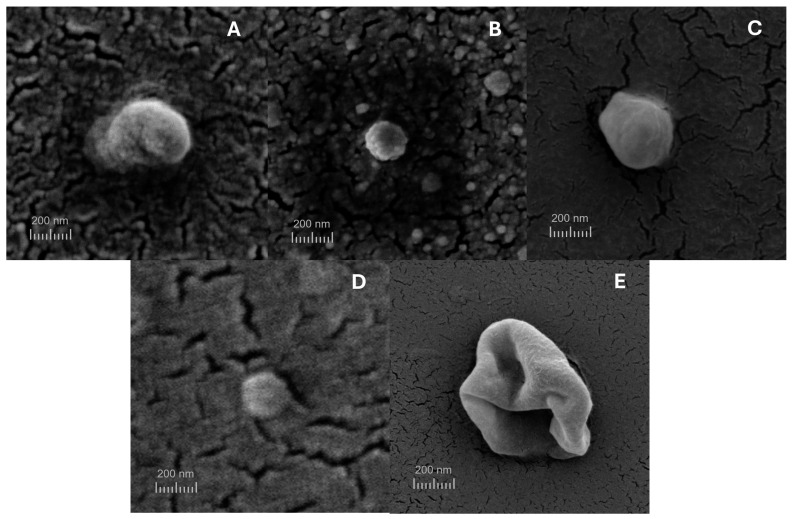
SEM images of (**A**) oNLC, (**B**) oNLC-Plu, (**C**) oNLC-Chol, (**D**) oNLC-PEG, and (**E**) oNLC-Chi.

The size and shape of nanoparticles are critical in determining various properties, including encapsulation efficiency and the cellular uptake of the encapsulated ingredients [51]. The NLC produced displayed a uniform spherical morphology with no detectable aggregates when analyzed using scanning electron microscopy (SEM), as illustrated in Figure 7, except for oNLC-Chi, which presented near-spherical particles with relatively smooth surfaces. This observation aligns with the size and PdI findings of the chitosan-functionalized formulations, as these did not exhibit suitable sizes (>651 nm) nor favorable macroscopic aspects, becoming easily destabilized. The spherical and homogeneous shape of the remaining NLCs indicates their suitability as efficient carriers for delivering bioactive compounds.

### 3.6. Quantification and Encapsulation Efficiency (EE%)

The calibration curves for carvacrol were constructed by plotting the peak areas against various concentrations. The resulting equation was y = 10066x + 3667.4, with R^2^ = 0.995. The high R^2^ values (>0.99) confirm the strong linearity of the method, demonstrating its reliability for quantifying the analytes. This is essential for accurately assessing variations in the concentration of the oil encapsulated within the carriers. Additionally, all formulations yielded quantification results closely aligned with the initial theoretical value (2% or 20 mg/mL), indicating a highly efficient encapsulation process with minimal carvacrol loss during NLC preparation. Encapsulation efficiency was consistently high across all formulations, ranging from 98.21% to 99.98%, signifying that the majority of carvacrol was successfully retained within the nanoparticles. Notably, the oNLC-Chol formulation exhibited the highest encapsulation efficiency. These findings suggest that all formulations are well-suited for the controlled delivery of carvacrol.

### 3.7. Stability Analysis

**Figure 8:**
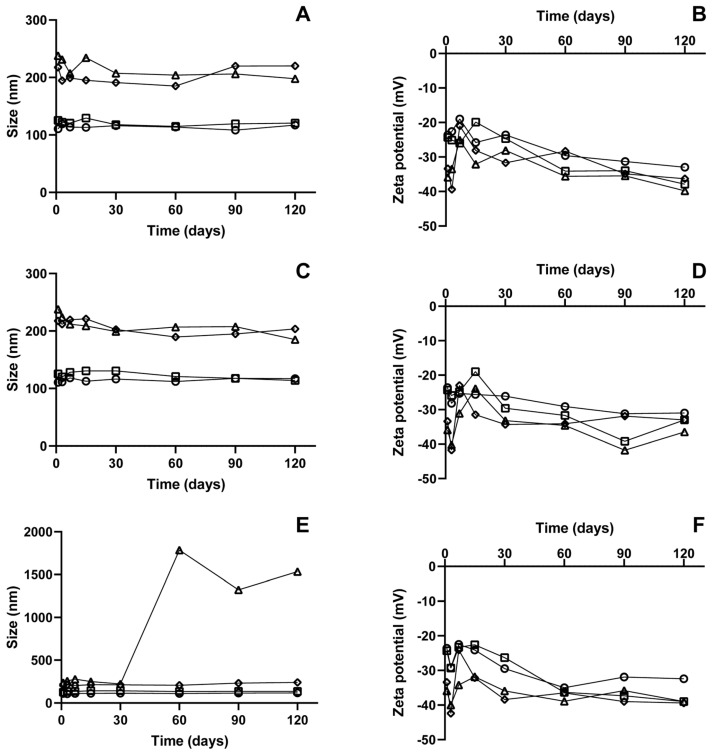
Particle size (**A**–**C**) and Zeta potential (**B**,**D**,**F**) of the formulations under different storage temperatures: 4 °C (**A**,**B**), 25 °C (**C**,**D**), and 37 °C (**E**,**F**) over 120 days. Chitosan-functionalized particles (oNLC-Chi0.05%) exhibited sizes outside the nanoscale range over time, indicating system instability and, therefore, their analysis was not continued. oNLC (**−O−**), oNLC-Plu0.5% (**−Δ−**), oNLC-Chol0.1% (**−☐−**), and oNLC-PEG0.5% (**−◊−**).

**Figure 9:**
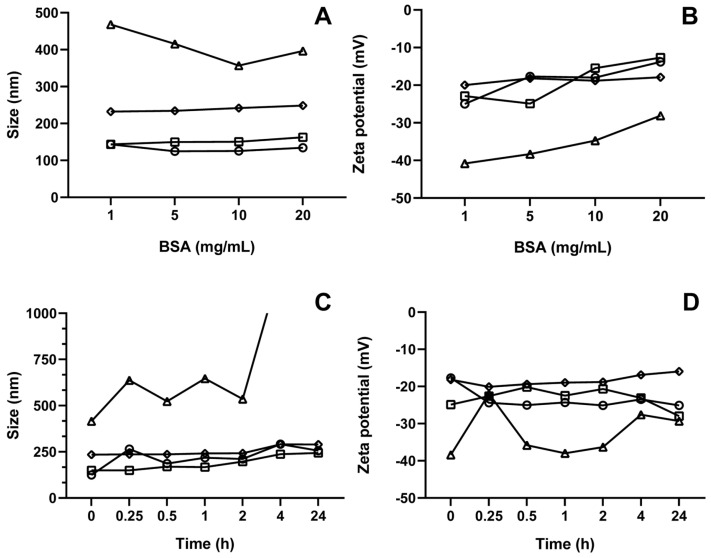
Particle size (**A**,**C**) and zeta potential (**B**,**D**) of NLCs after albumin corona formation at different concentrations (**A**,**B**) and over time (**C**,**D**). oNLC (**−O−**), oNLC-Plu (**−Δ−**), oNLC-Chol/10 (**−☐−**), and oNLC-PEG (**−◊−**).

Obtaining nanocarriers with optimized parameters that remain stable over time is crucial for the successful delivery of the active pharmaceutical ingredient, ensuring the intended therapeutic application. They may undergo chemical and physical degradation, potentially leading to reduced efficacy due to a decrease in formulation quality [52]. In this study, the optimized formulation and those functionalized with cholesterol (0.1%), Pluronic (0.5%), chitosan (0.05%), and PEG (0.5%) were evaluated in terms of size, polydispersity index (PdI), and zeta potential over 120 days at refrigerated (4 °C) (Figure 8A and Figure 9B), room temperature (25 °C) (Figure 8C,D), and incubator (37 °C) (Figure 8E,F).

When functionalized with cholesterol, the nanocarriers showed small variations in size, PdI, and zeta potential over 120 days for all temperatures, with no observable changes in their macroscopic appearance. Studies indicate that the cholesterol concentration employed in the system is critical to improving the lipid layer’s stability and maintaining integrity during a long period of storage. However, elevated cholesterol concentrations can increase the tendency for the systems to fuse during storage, resulting in larger particles [53]. These results highlight that the constituent concentrations used in this formulation were ideal for achieving a stable system over time and at varying temperatures.

The formulations functionalized with Pluronic did not exhibit significant changes in size, PdI, or zeta potential over time at 4 °C. However, this was not observed for samples stored at 25 °C and 37 °C, where destabilization began after three days of analysis. Macroscopic alterations were also noted, including flocculation and adhesion of the lipid material to the glass container. Variations in zeta potential could explain the aggregation of these particles, as they influence the stability of the nanocarrier. By the third day of analysis, these samples showed a slight increase in surface charge potential. Some studies suggest that increasing the concentration of Pluronic as a surfactant may impact formulation stability, with unabsorbed micelles potentially leading to flocculation. Particles functionalized with Pluronic tend to have a larger average diameter, and this size increase results in particles with lower electrokinetic potential, as this is directly proportional to the charge of the electrokinetic unit and decreases with increasing particle radius [54].

PEG is widely used as a functionalizing agent since it provides good colloidal stability to nanocarriers. PEG-functionalized nanoparticles repel each other, preventing molecular bonding due to steric hindrance [55]. PEG-functionalized formulations showed excellent particle size and PdI across all three analyzed temperatures, remaining between 218 and 241 nm, with a PdI between 0.25 and 0.31 over the 120-day analysis. However, after 15 days of testing, the formulations stored at 4 °C began to show macroscopic changes, as did the samples stored in the incubator after 60 days. PEG is the most commonly used macromolecule to extend the half-life of nanocarriers and significantly impacts nanoparticle structure, stabilization, and biodistribution in both in vitro and in vivo environments [55]. The results of our work demonstrated that PEG acted as a good functionalization agent for the system. However, some factors crucial to maintaining the system’s stability, such as the interaction between formulation constituents, may have been affected over time, resulting in macroscopic changes in these samples, even though particle size, PdI, and zeta potential did not exhibit significant alterations.

The formulation functionalized with chitosan showed a particle size outside the range presented by the other nanocarrier systems, with an average size of 651 nm, which gradually increased over time. Conventional synthesis of nanoparticles with chitosan often results in large particles or the formation of aggregates of smaller particles due to chitosan’s mucoadhesive properties. Methodologies described in the literature for obtaining these nanocarriers present limitations, particularly related to controlling the size of these nanoparticles. Factors such as the pH of the medium, fractions of free primary amino groups, and solute concentration can be critical to the stability and formation of these nanoparticles [56]. Therefore, the functionalized nanocarriers with chitosan, which averaged 651 nm, likely resulted from low interaction between the chemical groups of the formulation’s other constituents and chitosan, as well as the low stability due to chitosan’s mucoadhesive characteristics and its easy degradability in the medium. This favored the formation of aggregates between smaller nanoparticles and probable degradation products of chitosan.

### 3.8. Corona Formation Evaluation

When a nanomaterial is exposed to biological fluids, layers of proteins and/or biomolecules can form around it, known as the protein corona, which has a substantial influence on various biological processes, such as biodistribution, particle stability, and immune system recognition [57]. The particle size, NLC composition, and surface charge can affect the type and quantity of protein attracted [58].

Surface modification of nanoparticles can influence the type of protein corona formed. For example, functionalization with stealth materials like PEG can minimize protein adsorption, thereby reducing opsonization and prolonging circulation time in the bloodstream [58]. Moreover, if system destabilization does not occur, the formation of a protein corona should not be considered entirely detrimental, as tumor cells tend to overexpress albumin receptors such as gp60. This can assist in better targeting of coated particles to the target tissue, greater accumulation in the tumor microenvironment, increased cellular uptake, and improved therapeutic efficacy [59].

As illustrated in Figure 9, the variations in particle size and zeta potential following protein corona formation were assessed based on albumin concentration and incubation time. Figure 9A,B depict the immediate changes in particle size and zeta potential, respectively, across all formulations at different albumin concentrations (1, 5, 10, and 20 mg/mL) after incubation. In contrast, Figure 9C,D present the temporal changes in particle size and zeta potential, respectively, for all formulations at a fixed BSA concentration of 5 mg/mL. It is important to note that oNLC-Chi was not included in the analysis due to the previously discussed destabilization.

When incubated at varying albumin concentrations, the particle size remained relatively stable across the majority of formulations, except for oNLC-Plu, which showed an increase in size, resulting in destabilization and increased PdI at all BSA concentrations (357 to 469 nm) (Figure 9A). This can be attributed to albumin molecules adsorbing onto the nanoparticle surface according to their levels of hydrophobicity and electrostatic charge [60], increasing in particle size as protein molecules occupy available space on the surface. The zeta potential also became less negative as more BSA was added, indicating a reduction in the magnitude of surface charge for all formulations, precisely due to the weakly negative charge of albumin now coating the particles (58) (Figure 9B).

Furthermore, incubation time had no significant impact on particle size (Figure 9C), except again for oNLC-Plu1%, which exhibited immediate destabilization following incubation (416 nm), and oNLC-Chol, which showed a slight increase in size (244 nm) after 24 h. This increase in size over time may occur because albumin adsorption is dynamic, meaning the accumulation of protein mass leads to a structural reorganization of albumin molecules within the initially adsorbed layer, forming a multilayer, which increases the corona diameter [61]. Additionally, the protein corona can continuously rearrange and mature upon interaction with proteins. This dynamic process may facilitate the formation of molecular bridges between albumin and the coated nanoparticles, especially with hydrophilic polymers like Pluronic, leading to the formation of larger clusters or aggregates [57,62].

For zeta potential (Figure 9D), there were small variations over time, except for oNLC-PEG, which showed a more significant decrease in zeta potential, remaining below −20 mV (−16 to −20 mV). Despite these changes, they did not impact the overall charge or stability of NLCs.

### 3.9. Cytotoxicity Assay

The cytotoxicity of oNLC, oNLC MCT, oNLC-Plu0.05%, oNLC-Chol0.1%, oNLC-PEG0.5%, and carvacrol was evaluated in the MCF-7 cell line. The results are presented in Table 3, which shows the concentration required to inhibit 50% of cell viability (IC_50_) for the sample after 24 h of treatment.

**Figure 10:**
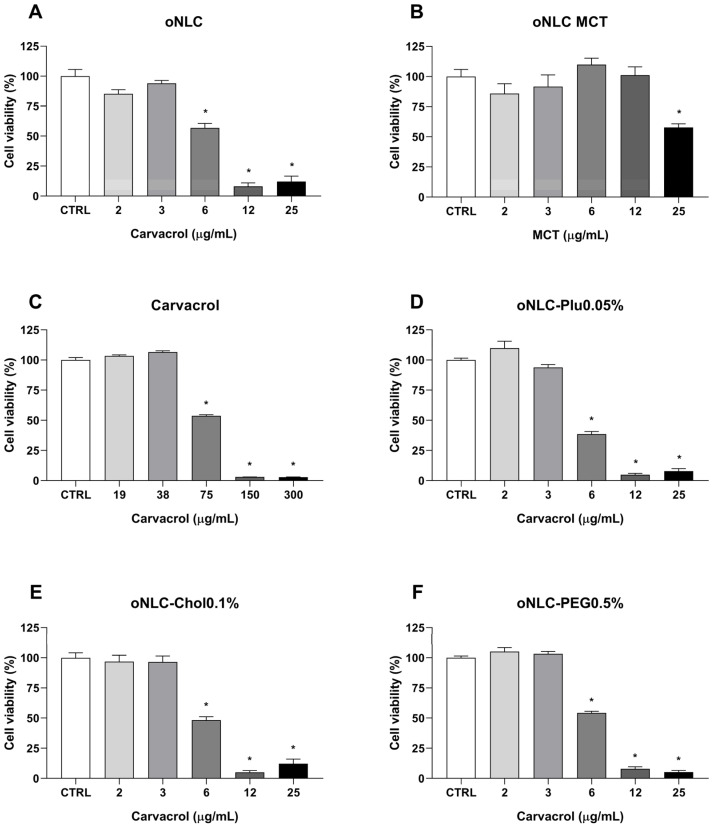
Cytotoxicity in the MCF-7 cell line after treatment with (**A**) oNLC, (**B**) oNLC MCT, (**C**) carvacrol, (**D**) oNLC-Plu0.05%, (**E**) oNLC-Chol0.1%, and (**F**) oNLC-PEG0.5%. * CTRL: negative control.

As observed, carvacrol presented an IC_50_ value of 77 ± 1 μg/mL for the MCF-7 cell line after 24 h of treatment. Incorporation of this oil into NLCs increased its cytotoxic effect by 12-fold, resulting in an IC_50_ of 7 ± 1 μg/mL for the oNLC sample, while the blank formulation (oNLC MCT) had an IC_50_ higher than 25 μg/mL. For the other formulations, the IC_50_ ranged between 7 and 6 μg/mL, with the lowest value obtained for oNLC-Plu 0.05% (6 ± 0.4 μg/mL). These results are also presented in Figure 10.

It was observed that the oNLC formulation significantly reduced cell viability starting at a concentration of 6 μg/mL, with an inhibition of 43 ± 4% (*p* < 0.001) compared to the control group. On the other hand, carvacrol (Figure 10C) showed a significant reduction in cell viability only at 75 μg/mL, with an inhibition of 46 ± 1% (*p* < 0.001). The other formulations (Figure 10D–F) presented similar cytotoxic profiles to oNLC, with a significant reduction in cell viability compared to the control group, starting at 6 μg/mL. Notably, the oNLC-Plu 0.05% formulation reduced viability by 61 ± 2% (*p* < 0.001) at this concentration. Figure 10B represents the blank formulation (oNLC MCT), which only at a concentration of 25 μg/mL significantly reduced cell viability compared to the control group (inhibition percentage of 42 ± 3%; *p* < 0.001). The data (Supplementary Materials, Table S1) demonstrate that all formulations and carvacrol showed lower cytotoxicity in the non-tumoral MCF-10 cell line compared to the MCF-7 breast cancer cell line. At a concentration of 7 μg/mL, the maximum IC_50_ value for nanoparticles, cell viability in MCF-10 cells ranged from 96% to 123%, suggesting a potential selectivity for tumor cells (MCF-7). This observed selectivity highlights the ability of the formulations to preferentially target cancer cells, sparing normal cells. These results revealed that oNLCs significantly enhanced the anticancer activity of carvacrol, demonstrating the effectiveness of nanoformulations in improving drug delivery and reducing the required therapeutic dose, addressing a key limitation of conventional carvacrol treatments.

## 4. Conclusions

Nanostructured lipid carriers containing carvacrol were produced and functionalized to enhance the cytotoxicity of carvacrol against breast cancer cells. The optimized NLC was successfully functionalized with Pluronic^®^, cholesterol, and PEG at varying concentrations, leading to modifications in particle size and zeta potential while maintaining stability for 120 days at 4 °C. The NLC functionalized with chitosan rapidly destabilized after production. All other NLC formulations exhibited a uniform spherical morphology and demonstrated good stability over time and with increasing albumin concentrations upon incubation, except for oNLC-Plu1%, which underwent immediate destabilization, and oNLC-Chol, which experienced a slight increase in size after 24 h. Finally, the incorporation of carvacrol into oNLC increased its cytotoxic effect by 12-fold, resulting in an IC_50_ of 7 ± 1 μg/mL. Thus, this nanotechnology system, functionalized and designed with compatible features, promises future applications in breast cancer treatment.

## Figures and Tables

**Table 1:** Particle size, polydispersity index (PdI), and zeta potential of pre-formulations (PFs) produced with different concentrations of surfactants (Lipoid S-100 (-L), Kolliphor ELP (-K) and Span 60 (-S)).

NLC	Carvacrol (%)	Precirol^®^ (%)	Lipoid^®^ S-100 (%)	Kolliphor^®^ ELP (%)	Span^®^ 60 (%)	Size (nm)	PdI	Zeta Potential (mV)
PF01-L	2	1	1	-	-	273 ± 2	0.41 ± 0.03	−21 ± 1
PF01-K	2	1	-	1	-	173 ± 1	0.15 ± 0.01	−18 ± 1
PF01-S	2	1	-	-	1	115 ± 1	0.25 ± 0.01	−24 ± 1
PF02-L	5	4	1	-	-	289 ± 6	0.40 ± 0.08	−36 ± 0.2
PF02-K	5	4	-	1	-	224 ± 3	0.24 ± 0.01	−20 ± 0.1
PF02-S	5	4	-	-	1	208 ± 5	0.25 ± 0.01	−22 ± 1
PF03-L	9	7	1	-	-	310 ± 4	0.35 ± 0.02	−29 ± 1
PF03-K	9	7	-	1	-	300 ± 1	0.33 ± 0.01	−24 ± 0.4
PF03-S	9	7	-	-	1	283 ± 2	0.24 ± 0.01	−30 ± 0.5

**Table 2:** Average size, polydispersity index, and zeta potential found for the formulations produced using Span 60 (1%) as the lipophilic surfactant.

	Variables			Size (nm)	PdI	Zeta Potential (mv)
NLC	Carvacrol (%)	Precirol^®^ (%)	Tween^®^ 80 (%)			
1	5	1	1	220 ± 9	0.24 ± 0.04	−36 ± 1
2	5	4	3	248 ± 7	0.23 ± 0.02	−28 ± 0.2
3	5	1	5	208 ± 6	0.25 ± 0.04	−22 ± 1
4	9	4	5	283 ± 2	0.24 ± 0.01	−30 ± 1
5	9	4	1	333 ± 12	0.10 ± 0.07	−34 ± 1
6	1	4	5	109 ± 2	0.24 ± 0.03	−18 ± 0.4
7	5	4	3	240 ± 9	0.24 ± 0.03	−28 ± 0.1
8	1	1	3	88 ± 2	0.30 ± 0.01	−19 ± 1
9	1	7	3	309 ± 12	0.47 ± 0.06	−24 ± 2
10	9	1	3	263 ± 2	0.24 ± 0.03	−39 ± 1
11	5	7	5	251 ± 12	0.47 ± 0.05	−20 ± 1
12	1	4	1	263 ± 12	0.49 ± 0.06	−11 ± 1
13	5	7	1	567 ± 24	0.21 ± 0.03	−24 ± 1
14	5	4	3	250 ± 8	0.22 ± 0.04	−32 ± 0.3
15	9	7	3	289 ± 1	0.05 ± 0.07	−28 ± 1

**Table 3:** IC_50_ in MCF-7 after treatment with oNLC, oNLC MCT, oNLC-Plu0.05%, oNLC-Chol0.1%, oNLC-PEG0.5%, and carvacrol for 24 h by MTT assay.

NLCs	IC_50_ (μg/mL)
Carvacrol	77 ± 1
oNLC	7 ± 1
oNLC MCT	>25
oNLC-Plu0.05%	6 ± 0.4
oNLC-Chol0.1%	6 ± 0.4
oNLC-PEG0.5%	7 ± 0.3

## Data Availability

The original contributions presented in the study are included in the article Supplementary Materials; further inquiries can be directed to the corresponding author.

## Supplementary Materials

The following supporting information can be downloaded at https://www.mdpi.com/article/10.3390/pharmaceutics17030363/s1: Figure S1. Carvacrol (black line) and oNLC (Grey line) chromatograms (A) and calibration curve of carvacrol (B); Figure S2. Particle size resulting from increased carvacrol concentrations; Table S1. The cytotoxicity of Carvacrol, oNCL, oNCL MCT, oNCL-Plu0.05%, oNCL-Chol0.1%, and oNCL-PEG0.5%, in the MCF-10 cell line after 24 h of treatment assessed by the MTT assay.

## Abbreviations

The following abbreviations are used in this manuscript:

NLC	Nanostructured lipid carrier
BSA	Bovine serum albumin
DLS	Dynamic light scattering
DMEM	Dulbecco’s Modified Eagle Medium
DMSO	Dimethyl sulfoxide
EE%	Encapsulation efficiency
FTIR	Fourier-transform infrared microscopy
HES	Hot emulsification followed by sonication
PBS	Phosphate-buffered saline
PdI	Polydispersity index
PEG	Polyethylene glycol
SDS	Sodium dodecyl sulfate
MCT	Medium-chain triglyceride
MTT	3-(4,5-dimethylthiazol-2-yl)-2,5-diphenyltetrazolium bromide

## References

[1] Siegel R.L., Miller K.D., Jemal A. (2020). Cancer statistics, 2020. CA A Cancer J Clin..

[2] Brown J.S., Amend S.R., Austin R.H., Gatenby R.A., Hammarlund E.U., Pienta K.J. (2023). Updating the definition of cancer. Mol. Cancer Res..

[3] Al Saqr A., Wani S.U.D., Gangadharappa H.V., Aldawsari M.F., Khafagy E.S., Lila A.S.A. (2021). Enhanced cytotoxic activity of docetaxel-loaded silk fibroin nanoparticles against breast cancer cells. Polymers.

[4] Berko Y.A., Funmilola A.F., Akala E.O. (2021). Fabrication of paclitaxel and 17aag-loaded poly-ε-caprolactone nanoparticles for breast cancer treatment. J. Pharm. Drug Deliv. Res..

[5] Sung H., Ferlay J., Siegel R.L., Laversanne M., Soerjomataram I., Jemal A., Bray F. (2021). Global cancer statistics 2020: Globocan estimates of incidence and mortality worldwide for 36 cancers in 185 countries. CA A Cancer J. Clin..

[6] Tagde P., Najda A., Nagpal K., Kulkarni G.T., Shah M., Ullah O., Balant S., Rahman H. (2022). Nanomedicine-based delivery strategies for breast cancer treatment and management. Int. J. Mol. Sci..

[7] Castillo-Tobías I., Berlanga L., Poblano J., Rodríguez-Salazar M.D.C., Aguayo-Morales H., Cobos-Puc L.E. (2023). Fundamental considerations of targeted drug therapies for breast cancer. Future Pharmacol..

[8] Prayoga D.K., Aulifa D.L., Budiman A., Levita J. (2024). Plants with anti-ulcer activity and mechanism: A review of preclinical and clinical studies. Drug Des. Devel Ther..

[9] Capatina L., Napoli E.M., Ruberto G., Hritcu L. (2021). *Origanum vulgare* ssp. Hirtum (Lamiaceae) essential oil prevents behavioral and oxidative stress changes in the scopolamine zebrafish model. Molecules.

[10] Sampaio L.A., Pina L.T.S., Serafini M.R., Tavares D.D.S., Guimarães A.G. (2021). Antitumor effects of carvacrol and thymol: A systematic review. Front. Pharmacol..

[11] Moghrovyan A., Parseghyan L., Sevoyan G., Darbinyan A., Sahakyan N., Gaboyan M., Karabekian Z., Voskanyan A. (2022). Antinociceptive, anti-inflammatory, and cytotoxic properties of *Origanum vulgare* essential oil, rich with β-caryophyllene and β-caryophyllene oxide. Korean J. Pain..

[12] Avola R., Granata G., Geraci C., Napoli E., Graziano A.C.E., Cardile V. (2020). Oregano (*Origanum vulgare* L.) essential oil provides anti-inflammatory activity and facilitates wound healing in a human keratinocytes cell model. Food Chem. Toxicol..

[13] Abdollahi B.B., Malekzadeh R., Azar F.P., Salehnia F., Naseri A.R., Ghorbani M., Hamishehkar H., Farajollahi A.R. (2020). Main approaches to enhance radiosensitization in cancer cells by nanoparticles: A systematic review. Adv. Pharm. Bull..

[14] Ombredane A.S., Silva V.R.P., Andrade L.R., Pinheiro W.O., Simonelly M., Oliveira J.V., Pinheiro A.C., Gonçalves G.F., Felice G.J., Garcia M.P. (2021). In vivo efficacy and toxicity of curcumin nanoparticles in breast cancer treatment: A systematic review. Front. Oncol..

[15] Hamimed S., Jabberi M., Chatti A. (2022). Nanotechnology in drug and gene delivery. Naunyn Schmiedebergs Arch. Pharmacol..

[16] Gomaa E., Fathi H.A., Eissa N.G., Elsabahy M. (2022). Methods for preparation of nanostructured lipid carriers. Methods.

[17] Patel V.R., Agrawal Y.K. (2011). Nanosuspension: An approach to enhance solubility of drugs. J. Adv. Pharm. Technol. Res..

[18] Makeen H.A., Mohan S., Al-Kasim M.A., Sultan M.H., Albarraq A.A., Ahmed R.A., Alhazmi H.A., Alam M.I. (2021). Preparation, Characterization, and Anti-Cancer Activity of Nanostructured Lipid Carriers Containing Imatinib. Pharmaceutics.

[19] Uchôa A.F.C., Formiga A.L.D., Alves Á.E.F., Cardoso A.L.M.R., Pereira G.M.d.A., Carvalho L.M.M., da Silva L.F.A., Pereira P.S.d.S., de Souza P.H.O., Jales S.T.L. (2025). Optimization and Functionalization of Copaiba Oil-Loaded Nanostructured Lipid Carriers to Improve Cytotoxicity against Breast Cancer Cells. J. Drug Deliv. Sci. Technol..

[20] Mura P., Maestrelli F., D’Ambrosio M., Luceri C., Cirri M. (2021). Evaluation and comparison of solid lipid nanoparticles (Slns) and nanostructured lipid carriers (Nlcs) as vectors to develop hydrochlorothiazide effective and safe pediatric oral liquid formulations. Pharmaceutics.

[21] Danaei M., Dehghankhold M., Ataei S., Hasanzadeh Davarani F., Javanmard R., Dokhani A., Khorasani S., Mozafari M.R. (2018). Impact of particle size and polydispersity index on the clinical applications of lipidic nanocarrier systems. Pharmaceutics.

[22] Tazehjani D.A.J., Farahpour M.R., Hamishehkar H. (2021). Effectiveness of topical caraway essential oil loaded into nanostructured lipid carrier as a promising platform for the treatment of infected wounds. Colloids Surf. A Physicochem. Eng. Asp..

[23] Subramaniam B., Siddik Z.H., Nagoor N.H. (2020). Optimization of nanostructured lipid carriers: Understanding the types, designs, and parameters in the process of formulations. J. Nanopart. Res..

[24] Gavini E., Rassu G., Sanna V., Cossu M., Giunchedi P. (2010). Mucoadhesive microspheres for nasal administration of an antiemetic drug, metoclopramide: In-vitro/ex-vivo studies. J. Pharm. Pharmacol..

[25] Trivino A., Gumireddy A., Chauhan H. (2019). Drug-lipid-surfactant miscibility for the development of solid lipid nanoparticles. AAPS PharmSciTech.

[26] da Silva G.H.R., de Moura L.D., de Carvalho F.V., Geronimo G., Mendonça T.C., de Lima F.F., de Paula E. (2021). Antineoplastics encapsulated in nanostructured lipid carriers. Molecules.

[27] Vinsova J., Vavrikova E. (2008). Recent advances in drugs and prodrugs design of chitosan. Curr. Pharm. Des..

[28] Valencia M.S., Júnior M.F.d.S., Júnior F.H.X., Veras B.d.O., Borba E.F.d.O., da Silva T.G., Xavier V.L., de Souza M.P., Carneiro-Da-Cunha M.d.G. (2021). Bioactivity and cytotoxicity of quercetin-loaded, lecithin-chitosan nanoparticles. Biocatal. Agric. Biotechnol..

[29] Jana P., Shyam M., Singh S., Jayaprakash V., Dev A. (2021). Biodegradable polymers in drug delivery and oral vaccination. Eur. Polym. J..

[30] Pyo Y.C., Tran P., Kim D.H., Park J.S. (2020). Chitosan-coated nanostructured lipid carriers of fenofibrate with enhanced oral bioavailability and efficacy. Colloids Surf. B Biointerfaces.

[31] Kontogiannis O., Selianitis D., Perinelli D.R., Bonacucina G., Pippa N., Gazouli M., Pispas S. (2022). Non-ionic surfactant effects on innate pluronic 188 behavior: Interactions, and physicochemical and biocompatibility studies. Int. J. Mol. Sci..

[32] Sek L., Boyd B.J., Charman W.N., Porter C.J.H. (2006). Examination of the impact of a range of Pluronic surfactants on the in-vitro solubilisation behaviour and oral bioavailability of lipidic formulations of atovaquone. J. Pharm. Pharmacol..

[33] Sun Y., Wang Q., Chen J., Liu L., Ding L., Shen M., Li J., Han B., Duan Y. (2017). Temperature-sensitive gold nanoparticle-coated pluronic-pll nanoparticles for drug delivery and chemo-photothermal therapy. Theranostics.

[34] Zhao S., Yang X., Garamus V.M., Handge U.A., Bérengère L., Zhao L., Salamon G., Willumeit R., Zou A., Fan S. (2014). Mixture of nonionic/ionic surfactants for the formulation of nanostructured lipid carriers: Effects on physical properties. Langmuir.

[35] Chen Y., Zhu Y., Li X., Gao W., Zhen Z., Dong D., Huang B., Ma Z., Zhang A., Song X. (2022). Cholesterol inhibits TCR signaling by directly restricting TCR-CD3 core tunnel motility. Mol. Cell.

[36] Kuo Y., Wang C. (2014). Cationic solid lipid nanoparticles with cholesterol-mediated surface layer for transporting saquinavir to the brain. Biotechnol. Prog..

[37] Ramalingam P., Ko Y.T. (2015). Enhanced oral delivery of curcumin from n-trimethyl chitosan surface-modified solid lipid nanoparticles: Pharmacokinetic and brain distribution evaluations. Pharm. Res..

[38] Abumanhal-Masarweh H., da Silva D., Poley M., Zinger A., Goldman E., Krinsky N., Kleiner R., Shenbach G., Schroeder J.E., Shklover J. (2019). Tailoring the lipid composition of nanoparticles modulates their cellular uptake and affects the viability of triple negative breast cancer cells. J. Control. Release.

[39] Karn-Orachai K., Smith S.M., Phunpee S., Treethong A., Puttipipatkhachorn S., Pratontep S., Ruktanonchai U.R. (2014). The effect of surfactant composition on the chemical and structural properties of nanostructured lipid carriers. J. Microencapsul..

[40] Gardouh A.R., Faheim S.H., Noah A.T., Ghorab M.M. (2018). Influence of formulation factors on the size of nanostructured lipid carriers and nanoemulsions prepared by high shear homogenization. Int. J. Pharm. Pharm. Sci..

[41] Hoang Thi T.T., Pilkington E.H., Nguyen D.H., Lee J.S., Park K.D., Truong N.P. (2020). The importance of poly(Ethylene glycol) alternatives for overcoming peg immunogenicity in drug delivery and bioconjugation. Polymers.

[42] Suk J.S., Xu Q., Kim N., Hanes J., Ensign L.M. (2016). PEGylation as a strategy for improving nanoparticle-based drug and gene delivery. Adv. Drug Deliv. Rev..

[43] Hu C., Lei T., Wang Y., Cao J., Yang X., Qin L., Liu R., Zhou Y., Tong F., Umeshappa C.S. (2020). Phagocyte-membrane-coated and laser-responsive nanoparticles control primary and metastatic cancer by inducing anti-tumor immunity. Biomaterials.

[44] Kurczewska J. (2023). Chitosan-based nanoparticles with optimized parameters for targeted delivery of a specific anticancer drug—A comprehensive review. Pharmaceutics.

[45] Valderrama A.C.S., Rojas De G.C. (2017). Traceability of active compounds of essential oils in antimicrobial food packaging using a chemometric method by ATR-FTIR. Am. J. Anal. Chem..

[46] Yoncheva K., Benbassat N., Zaharieva M.M., Dimitrova L., Kroumov A., Spassova I., Kovacheva D., Najdenski H.M. (2021). Improvement of the antimicrobial activity of oregano oil by encapsulation in chitosan—Alginate nanoparticles. Molecules.

[47] Torchio A., Cassino C., Lavella M., Gallina A., Stefani A., Boffito M., Ciardelli G. (2021). Injectable supramolecular hydrogels based on custom-made poly(Ether urethane)s and α-cyclodextrins as efficient delivery vehicles of curcumin. Mater. Sci. Eng. C.

[48] Witika B.A., Walker R.B. (2021). Preformulation characterization and identification of excipients for nevirapine loaded niosomes. Pharmazie.

[49] Zhu H., Tang H., Li F., Sun H., Tong L. (2023). Effect of milling intensity on the properties of chitin, chitosan and chitosan films obtained from grasshopper. Int. J. Biol. Macromol..

[50] Jia X., Tan R., Peng B. (2022). Preparation and application of polyethylene glycol triazine derivatives as a chrome-free tanning agent for wet-white leather manufacturing. Environ. Sci. Pollut. Res..

[51] Sobczyński J., Bielecka G. (2019). Nanostructure lipid carriers. Nanoparticles in Pharmacotherapy.

[52] Figueiredo P., Lahtinen M.H., Agustin M.B., de Carvalho D.M., Hirvonen S., Penttilä P.A., Mikkonen K.S. (2021). Green fabrication approaches of lignin nanoparticles from different technical lignins: A comparison study. ChemSusChem.

[53] Hu Y., Hoerle R., Ehrich M., Zhang C. (2015). Engineering the lipid layer of lipid-PLGA hybrid nanoparticles for enhanced in vitro cellular uptake and improved stability. Acta Biomater..

[54] Fayad S.J. (2010). Obtenção e Caracterização de Micro e Nanopartículas a Base de Proteína Isolada de Soja [Obtaining and Characterizing Micro and Nanoparticles Based on Isolated Soy Protein]. Master’s Dissertation.

[55] Conde J., Dias J.T., GrazÃo V., Moros M., Baptista P.V., De La Fuente J.M. (2014). Revisiting 30 years of biofunctionalization and surface chemistry of inorganic nanoparticles for nanomedicine. Front. Chem..

[56] Masarudin M.J., Cutts S.M., Evison B.J., Phillips D.R., Pigram P.J. (2015). Factors determining the stability, size distribution, and cellular accumulation of small, monodisperse chitosan nanoparticles as candidate vectors for anticancer drug delivery: Application to the passive encapsulation of [^14^C]-doxorubicin. Nanotechnol. Sci. Appl..

[57] Tenzer S., Docter D., Kuharev J., Musyanovych A., Fetz V., Hecht R., Schlenk F., Fischer D., Kiouptsi K., Reinhardt C. (2013). Rapid formation of plasma protein corona critically affects nanoparticle pathophysiology. Nat. Nanotech.

[58] Rampado R., Crotti S., Caliceti P., Pucciarelli S., Agostini M. (2020). Recent advances in understanding the protein corona of nanoparticles and in the formulation of “stealthy” nanomaterials. Front. Bioeng. Biotechnol..

[59] Fu Q., Sun J., Zhang W., Sui X., Yan Z., He Z. (2009). Nanoparticle albumin-bound (Nab) technology is a promising method for anti-cancer drug delivery. Recent Patents Anti-Cancer Drug Discov..

[60] Ding H.M., Ma Y.Q. (2014). Computer simulation of the role of protein corona in cellular delivery of nanoparticles. Biomaterials.

[61] Shourni S., Javadi A., Hosseinpour N., Bahramian A., Raoufi M. (2022). Characterization of protein corona formation on nanoparticles via the analysis of dynamic interfacial properties: Bovine serum albumin—Silica particle interaction. Colloids Surf. A Physicochem. Eng. Asp..

[62] Yu Y., Luan Y., Dai W. (2022). Dynamic process, mechanisms, influencing factors and study methods of protein corona formation. Int. J. Biol. Macromol..

