# Supplementary material for: Optimized and Functionalized Carvacrol-Loaded Nanostructured Lipid Carriers for Enhanced Cytotoxicity in Breast Cancer Cells
Source: Pharmaceutics. 2025 Mar 13;17(3):363. doi: 10.3390/pharmaceutics17030363 (PMC11945829; doi:10.3390/pharmaceutics17030363)
Supplement: Supplementary file 1 [file pharmaceutics-17-00363-s001.zip › pharmaceutics-3405986-supplementary.pdf]

Optimized and Functionalized Carvacrol-Loaded Nanostructured Lipid Carriers for Enhanced Cytotoxicity in Breast Cancer Cells

Ana F. C. Uchôa [1], Allessya L. D. Formiga [1], Anny L. M. R. Cardoso [1], Graziela M. A. Pereira [1], Lucas M. M. Carvalho [1], Pedro H. O. Souza [1], Anauara L. Silva [2], Ramon R. M. Souza [3], Marianna V. Sobral [3], Marcelo S. Silva [2], José M. Barbosa-Filho [2] and Francisco H. Xavier-Júnior [1,*]

[1] Laboratory of Pharmaceutical Biotechnology (BioTecFarm), Department of Pharmaceutical Sciences, Federal University of Paraiba, Campus Universitário I, Castelo Branco III-Cidade Universitária, João Pessoa 58051-900, PB, Brazil; anauchoa@ltf.ufpb.br (A.F.C.U.); allessya.formiga@ltf.ufpb.br (A.L.D.F.); annyleticiamarinho@ltf.ufpb.br (A.L.M.R.C.); graziela.maria.araujo.pereira@academico.ufpb.br (G.M.A.P.); lucas.medeiros2@academico.ufpb.br (L.M.M.C.); phos@academico.ufpb.br (P.H.O.S.)

[2] Multiuser Characterization and Analysis Laboratory (LMCA), Institute of Research in Drugs and Medicines, Federal University of Paraiba, Campus Universitário I, Castelo Branco III-Cidade Universitária, João Pessoa 58051-900, PB, Brazil; anauaralima@ltf.ufpb.br (A.L.S.); marcelosobral@ltf.ufpb.br (M.S.S.); jbarbosa@ltf.ufpb.br (J.M.B.-F.)

[3] Oncopharmacology Laboratory (ONCOFAR), Institute of Research in Drugs and Medicines (IPeFarM), Federal University of Paraiba, Campus Universitário I, Castelo Branco III-Cidade Universitária, João Pessoa 58051-900, PB, Brazil; ramonsouza@ltf.ufpb.br (R.R.M.S.); mariannavbs@gmail.com (M.V.S.)

* Correspondence: fhxj@academico.ufpb.br

Supplementary material

The chromatogram obtained for carvacrol or nanoparticles containing carvacrol shows a sharp and well-defined peak with a retention time of 4.5 minutes, suggesting the purity of carvacrol and no peak interference by the nanoparticle components (Figure 1A). The calibration curve was constructed by plotting the peak areas against the concentrations of the injected carvacrol. The equation of the calibration curve obtained for carvacrol was y=24360x+12954, with an R^2 of 0.993. This linearity, expressed by the high R^2 value (>0.99), confirms the suitability of the method for quantifying the analytes over a wide concentration range. Such linearity is crucial to ensure that variations in the concentration of the active compounds encapsulated in the carriers can be accurately detected and quantified.

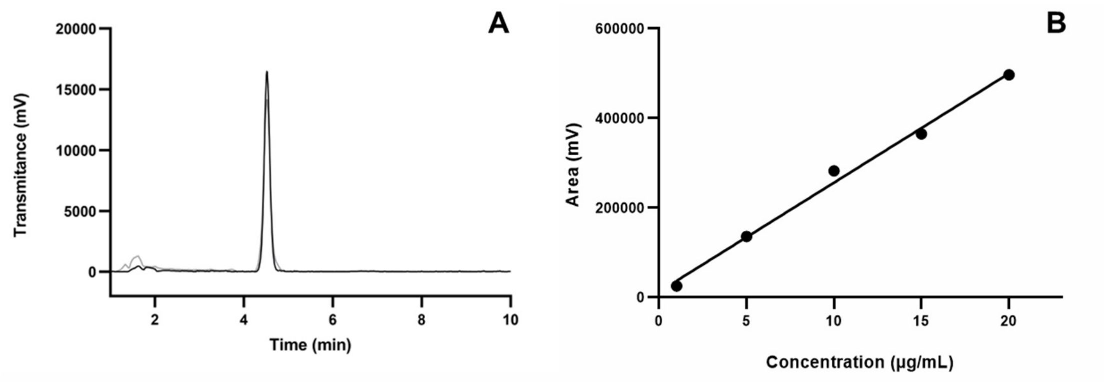

Figure S1. Carvacrol (black line) and oNLC (Grey line) chromatograms (A) and calibration curve of carvacrol (B)

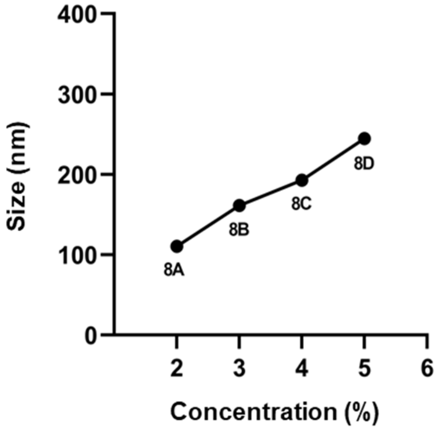

Figure S2. Particle size resulting from increased carvacrol concentrations

Concentration (µg/mL)	Cell viability (%)					
	Carvacrol	oNCL	oNCL MCT	oNCL-Plu0.05%	oNCL-Chol0.1%	oNCL-PEG0.5%
**1**	-	131 ± 5	90 ± 3	119 ± 4	102 ± 2	116 ± 3
**7**	-	121 ± 6	96 ± 2	118 ± 3	105 ± 3	123 ± 5
**40**	115 ± 4	4 ± 1	70 ± 1	6 ± 1	7 ± 1	5 ± 1
**75**	105 ± 6	-	-	-	-	-
**150**	4 ± 1	-	-	-	-	-

Table S1. The cytotoxicity of Carvacrol, oNCL, oNCL MCT, oNCL-Plu0.05%, oNCL-Chol0.1%, and oNCL-PEG0.5%, in the MCF-10 cell line after 24 hours of treatment assessed by the MTT assay. The data were obtained from an independent experiment performed in quadruplicate and are presented as the mean ± standard error of the mean (SEM) of the percentage of cell viability. MCT: Medium-chain triglycerides
